# A Much Needed Work

**Published:** 1886-04

**Authors:** 


### A MUCH NEEDED WORK.

### BIOGRAPHY OF TEXAS PHYSICIANS.

As it seems impossible to make all understand the object of the Biographical work now in progress, we wish to impress the main facts on the minds of our readers. From the force of circumstances,—early times and customs in Texas; it being then a frontier country, and (literally) infested with many bad characters—which gave a very *mixed* aspect to the population; its comparative isolation;—before the extension of railroads—the want of communication with the outside world; the absence, until comparatively late years, of organized medical societies; the absence of any medical publication, "Texas medicine" occupied unjustly, an obscure place in the annals of medicine in America. As Dr. Wilkinson expressed it, it was "an unknown quantity," "a terra incognito." Within the last two decades it has been redeemed from this obloquy, organized, dignified and made respectable; for it, recognition has been obtained amongst the profession at large, as the peer of that of any State or country.

The object of the Biography of Contemporary Physicians of Texas, now being written by the editor of this Journal is, to commemorate the lives of those physicians who have, by their services, the force of their characters, their upright and honorable walk in life, dignified, given character to, and made respectable the science and practice of medicine in the Lone Star State. It is meet and proper that this should be done, just as much so as that the political history of the State should be written; and the biographies of those of her sons who have shaped its destiny, and made it the Empire it is, politically, financially, and otherwise, and given to it its position of proud eminence amongst the nations of the earth, the peerless cotton and cattle State of the world, and foremost in the cause of education.

Medicine has advanced, side by side with other features of its civilization, and has developed *pari passu* with its wealth, population and railroad extensions; and those men who have devoted their lives to the consummation of that development, should go on record side by side with her statesmen, warriors, educators, jurists, financiers, agriculturists and merchants; justice demands it.

Texas is a young State, yet; civilization is recent; the impress of savage domination is scarcely obliterated; that of border times and "frontier" existence is not, altogether. These men—just alluded to, may be called the pioneers;—they have but laid the foundation of a great career. The history of Texas *begins* from the present,—and is comprehended within the last half century. Record of her development and progress, of her greatness, dates from the recent past,—her history is yet to be made and written. The present epoch bears the same relation to the future of the State as the struggle for independence, and the early colonial times of America do to the history of America. Surely, then, what has been done by way of beginning of a career, should be preserved; and the work and lives of all have been preserved except those of the physicians; and surely the medical profession have valid claims for recognition of their share in the work of shaping and of progress, as well as the others.

Medicine is yet in its infancy in Texas. In twenty years of God-given peace and prosperity to the country, our children will see, built upon the *foundation* laid by the pioneers and contemporary physicians of Texas, a superstructure of grand proportions. We will not see it; but those who come after us will enjoy the fruits of our labors and sacrifices. They will see a proud edifice arise—a temple of modern Esculapius,—a grand Medical Department of our grand State University;—and it will be peopled with modern Drakes, McDowells, Warrens, Yandells, Chapmans, Wistars, Hodges, Millers, Pancoasts and Grosses;- and a diploma from the Texas University will be a passport to the highest social, as well as professional privileges. They will see an able and venerable Texas Medical Journal grow up like a giant oak from the present little acorn, in whose heart there is the germ, which only requires the fostering care, and the genial support of an appreciative profession, to develop into future greatness and usefulness.

Let us, then, record the lives and services of those of Texas physicians who are now laying the foundation for this development.

